# Validation of ELPO-PT: A Risk Assessment Scale for Surgical Positioning Injuries in the Portuguese Context

**DOI:** 10.3390/nursrep14040236

**Published:** 2024-10-30

**Authors:** Andreia Salvini, Elsa Silva, Carmen Passos, Tânia Manuel, Camila Moraes, Clementina Sousa, Paulo Alves

**Affiliations:** 1 Nursing Research Unit IPO Research Center (CI-IPOP), Comprehensive Cancer Centre (Porto. CCC) & RISE@CI-IPOP (Health Research Network), 4200-072 Porto, Portugal; andreiasalvini@ipoporto.min-saude.pt; 2 Unidade Local de Saúde da Póvoa de Varzim/Vila do Conde (ULSPVVC), 4490-421 Póvoa do Varzim, Portugal; esilva@chpvvc.min-saude.pt; 3 Surgical Department—Operating Theater, Instituto Português Oncologia (IPO Porto), 4200-072 Porto, Portugal; carmenpassos68@gmail.com; 4 Centre for Interdisciplinary Research in Health|CIIS-Wounds Research Lab, Faculdade de Ciências da Saúde e Enfermagem, Universidade Católica Portuguesa, 4169-005 Porto, Portugal; taniamanuel@gruposaudenunomendes.pt; 5 PROVE.PT-Nuno Mendes Health Group, 4560-164 Penafiel, Portugal; 6 Nursing Institute, Universidade Federal do Rio de Janeiro, Rio de Janeiro 21941-902, Brazil; camila@macae.ufrj.br; 7 Nursing School, Instituto Politécnico de Viana do Castelo, Escola Superior de Saúde, 4900-347 Viana do Castelo, Portugal; clementinasousa@ess.ipvc.pt; 8 Health Sciences Research Unit—Nursing (UICISA:E), Escola Superior de Enfermagem de Coimbra, 3000-232 Coimbra, Portugal; 9 Faculty of Health Sciences and Nursing, Universidade Católica Portuguesa, 4169-005 Porto, Portugal; 10 Associação Portuguesa Tratamento de Feridas, 4420-283 Gondomar, Portugal

**Keywords:** nursing, perioperative nursing, intraoperative complications, assessment instrument, pressure ulcers

## Abstract

Background/Objectives: Surgical procedures carry inherent risks, including injuries from surgical positioning, which impact patient safety and healthcare quality. An instrument to assess and prevent these injuries is essential. This study aimed to validate and culturally adapt the ELPO-PT for the Portuguese population to ensure its applicability and effectiveness in assessing the risk of injury from surgical positioning. Methods: A validation study was conducted with 126 adult patients undergoing surgical procedures at a central hospital in northern Portugal. Statistical analyses, including the calculation of Cronbach’s alpha coefficient, assessed the internal reliability of the scale. Additionally, sensitivity and specificity analyses evaluated the ELPO-PT’s diagnostic accuracy in identifying patients at risk of developing positioning-related injuries. Results: The validation showed a Cronbach’s alpha coefficient of 0.782, indicating reasonable internal reliability. Sensitivity analysis revealed an 85% accuracy rate in identifying patients at risk of positioning injuries, while specificity analysis demonstrated a 90% accuracy rate for patients not at risk. Conclusions: The ELPO-PT is a valid and reliable instrument for aiding nurses in clinical decision-making, with significant sensitivity and specificity in identifying the risk of positioning-related injuries, including pressure ulcers, in adult patients during the intraoperative period. Its implementation is expected to be beneficial in healthcare settings, contributing to the prevention of complications associated with surgical positioning.

## 1. Introduction

Injuries resulting from surgical positioning represent a significant concern in the perioperative environment, leading to serious complications and the prolongment of patient recovery times. Improper positioning during surgery can cause a variety of injuries, including pressure ulcers, nerve damage, and musculoskeletal injuries [1]. Ensuring safety and preventing complications is the collective responsibility of the perioperative team [2]. Considering patients’ rights to safety and quality of care, the National Patient Safety Plan (PNSD, 2021–2026) calls on healthcare stakeholders (institutional managers, quality of care officials, patient safety officials, and risk management officials, as well as healthcare professionals, among others) to promote a culture of safety and to continuously implement safe practices. Effective communication is essential, especially during care transitions, transferals of responsibility, or informational exchanges among multidisciplinary team members, as these environments become increasingly complex. Surgical positioning is an essential component of perioperative nursing care, as it aims to prevent injuries and optimize patient outcomes [3].

Remember the following text:

Systematizing the decision-making process to manage risks associated with surgical positioning, such as pressure ulcers (PUs), nerve injuries, and perioperative pain, is essential to minimizing these conditions. The widely used Braden Scale, while effective for general PU risk assessment, lacks sensitivity in intraoperative settings.

In the absence of a specific tool in Portuguese for assessing the risk of developing PUs during surgery, the Risk Assessment Scale for the Development of Injuries Due to Surgical Positioning (ELPO) was adapted and validated for adult patients by Lopes et al. (2016) [4]. This study combines scientific evidence and the clinical need to validate a culturally adapted tool that supports healthcare professionals in systematizing their decision-making processes for managing PU risk during surgery.

Combining scientific evidence, interests, and the need for a validated instrument that improves the quality of care, the ELPO scale is a resource that attempts to systematize the decision-making process for professionals in managing the risk of PU development by providing objective indicators in this area and aiming at its prevention or minimization, considering its high incidence in this context.

The cultural adaptation and validation of risk assessment tools is a crucial step in ensuring the effectiveness of healthcare interventions across diverse populations [5,6]. This paper presents a methodological approach to the cultural adaptation and validation of the Risk Assessment Scale for the Development of Injuries Due to Surgical Positioning (ELPO), a tool designed to evaluate the risk of position-related injuries in surgical patients [5,7].

The initial development of the ELPO scale was based on a comprehensive review of intraoperative risk assessment tools [8]. Cultural differences can significantly impact the effectiveness of medical interventions, especially in high-stress situations like surgery. Therefore, it is essential that healthcare professionals possess cultural awareness to ensure optimal outcomes for all patients.

The adaptation process followed best practices, focusing on both surface-level and deep-level cultural components [6]. Involving the target population in the process was critical to ensuring the cultural appropriateness of the tool [9]. The validation process evaluated the psychometric properties of the ELPO scale, including reliability, construct validity, and predictive validity, to ensure its suitability for use in the Portuguese population. These findings will inform the development of culturally sensitive risk assessment tools in various healthcare settings.

Thus, the general objective of this study was to perform a cultural and linguistic adaptation of the ELPO measurement instrument to validate its use in the Portuguese population. Based on the formulation of this general objective, the following specific objectives were defined as follows:

- To translate the ELPO scale through cultural adaptation;
- To evaluate the conceptual and linguistic equivalences of the scale;
- To assess the scale’s psychometric properties;
- To evaluate the risk of developing injuries due to surgical positioning in patients undergoing surgery in various surgical specialties in the operating room.

This study aims to reduce the incidence and prevalence of PUs in the perioperative context, contributing to improved care for surgical patients. PUs have a significant impact on patient quality of life and healthcare resources. Preventing PUs also offers the potential for substantial institutional cost savings. By adapting and validating the ELPO scale for use in Portuguese contexts, this study contributes to advancing nursing practice by providing an effective tool for assessing PU risk in surgical patients, addressing a pressing clinical need.

In adapting and validating the ELPO, careful adherence to recommended guidelines and best practices was prioritized to ensure the instrument’s cultural equivalence for use in the Portuguese context. Previous studies have emphasized the importance of cultural adaptation and validation to ensure the quality and meaningful use of measurement instruments in diverse populations [10]. Previous adaptations of the ELPO scale have encountered several limitations and challenges that highlight the importance of cultural sensitivity in scale validation. One significant challenge was observed variation in surgical practice environments, including differences in the use of support surfaces and positioning techniques across different regions and healthcare systems. Additionally, patient populations varied substantially, particularly in terms of comorbidities and demographic factors, such as age, weight, and health conditions. These variations necessitate a careful and systematic approach when it comes to cultural adaptation, ensuring that the ELPO scale is both applicable and effective in assessing risk within the specific context of Portuguese healthcare settings. Addressing these challenges is critical to creating a reliable and valid version of the scale for local clinical practice.

The results of this study will provide valuable insights into the psychometric properties of the ELPO scale within the Portuguese context, including its reliability, construct validity, and predictive validity. These findings can inform the development of targeted interventions that mitigate the risk of position-related injuries in surgical patients, and that ultimately contribute to improving patient safety and quality of care [11,12].

## 2. Theoretical Framework

Pressure ulcers are among the most commonly reported iatrogenic adverse events, representing a significant problem due to the damaging impact they have on individuals and the high costs associated with their treatment [13,14]. They are one of the major issues addressed by organizational managers, given their high morbidity rate, risk of hospital-acquired infections, prolonged recovery time, and detrimental effect on patient quality of life [15]. According to the literature, pressure ulcers can be avoided in up to 95% of cases through the implementation of effective preventive measures [16]. Moreover, other technologies aimed at PU prevention are now being developed and tested, including new support surfaces and wound dressings [17,18,19].

Several studies have identified various risk factors for pressure ulcer development in surgical patients, including older age, malnutrition, immobility, sensory impairment, and poor circulation [20,21].

The Risk Assessment Scale for the Development of Injuries Due to Surgical Positioning (ELPO) was developed to address the need for a reliable and valid instrument for the assessment of the risks associated with position-related injuries in surgical patients. Its domains assess factors such as patient characteristics, surgical positioning, and intraoperative risk factors. The scientific literature describes over a hundred risk factors for the development of intraoperative PUs, demonstrating the complexity of this phenomenon [4,22,23]. According to these authors, the operating room is a high-incidence and high-prevalence location for PUs due to the diversity and specificity of inherent risk factors, whether intrinsic to the patient or extrinsic (i.e., related to the perioperative environment). Adding to this, medical devices, while often essential for surgical procedures, inherently increase this risk by exerting localized pressure on vulnerable areas, potentially impeding blood flow and exacerbating the likelihood of PU development [24]. The site where the surgery takes place is where intrinsic and extrinsic risk factors converge.

In a study from 2018 [25], intraoperative pressure ulcers were defined as those that occur within several hours to 6 days after surgery. Similarly, another study from 2016 [4] found that 40% of patients experienced postoperative pain due to surgical positioning, and 21.7% developed pressure ulcers, with 12% already having Stage I lesions preoperatively, which then progressed to Stage II. The ELPO scale was developed to address the need for a tool that could systematically assess the risk associated with surgical positioning injuries.

According to some studies [25,26], surgical patients have a higher risk of developing pressure ulcers compared to non-surgical patients. This elevated prevalence is linked to several factors related to surgery, including surgical duration, fasting, post-anesthetic positioning, padding devices, moist skin, and skin preparation solutions.

The Braden Scale is a widely recognized and extensively reported on instrument in the scientific literature, with highest consensus globally, making it a commonly used tool for assessing the risk of pressure ulcer development in clinical practice. However, this scale does not adequately address the specific risk factors associated with the surgical patient population. Previous studies have demonstrated the need for a validated and culturally adapted instrument for the assessment of pressure ulcers risks and other positioning-related injuries in surgical patients [4,23,27,28,29,30].

However, a meta-analysis from 2012 [31] has suggested that the Braden Scale is not sensitive or effective in determining the risk of PU in surgical patients as it does not consider specific risk factors within the perioperative context, particularly those associated with the intraoperative period. The authors of this study recommend that this scale should not be used as the sole instrument for predicting PU risk in surgical patients, but rather that it should be used in the pre- and postoperative periods, complementing a specific instrument for assessing PU risk in the perioperative context.

Aiming to address this need, a specific tool for assessing the risk of PU development in the surgical context was developed and validated, known as the Risk Assessment Scale for the Development of Injuries Due to Surgical Positioning (ELPO) in adult patients [4]. The developers on this tool noted that it could guide clinical practice for nurses, aiding in their decision-making about patient care in the intraoperative period, particularly concerning surgical positioning.

Implementing evidence-based guidelines and interventions requires an accurate early assessment of each patient’s needs and risks. This is the only way to ensure that the implemented interventions will be effective, leading to nursing-sensitive health improvements, particularly in preventing pressure ulcers [32,33].

The development of the ELPO scale aims to fill an important gap in the assessment of positioning-related risks in surgical patients, complementing existing tools like the Braden Scale.

## 3. Materials and Methods

This study followed rigorous methodological guidelines for the cultural adaptation and validation of the ELPO scale. To adapt the ELPO scale to Portuguese culture and to validate it within the context of a central hospital, a quantitative, methodological, and cross-sectional study was designed.

### 3.1. Cultural Adaptation and Instrument Validation

The cross-cultural adaptation of the ELPO followed the principles recommended in the literature for adapting instruments across diverse linguistic and cultural contexts. The scale was translated and adapted according to the guidelines set out by Beaton et al. (2000) [34]. The adopted methodology closely followed the approach used by the original scale’s author to ensure the reproducibility and comparability of the results.

The process of culturally and linguistically adapting a scale involves the following steps: an initial translation by two independent translators, a synthesis of the two translated versions, a back-translation, and then a review by an expert panel [35]. This process of translation aims to make possible comparisons between concepts in the cultures of Brazil and Portugal.

The translation and cross-cultural adaptation procedure for the ELPO scale involved several sequential methodological steps. It began with formal requests for authorization from the author of the ELPO scale, the Administration Council of the hospital institution, and the Ethics Committee, all of which provided positive feedback.

The translation and cultural adaptation of the original instrument to European Portuguese involved the following processes (see Table 1 below):

### 3.2. Population and Sample

The study sample consisted of adult patients of both genders who underwent elective surgical procedures in any surgical specialty at a renowned and prestigious hospital in the north of Portugal. The selection considered convenience and accessibility factors as well as less-studied specifics, such as pressure ulcer development in oncology patients.

The sampling method was non-probabilistic and convenience-based, ensuring the inclusion criteria were met to promote homogeneity [38]. The inclusion and exclusion criteria for participant selection were:

- Inclusion Criteria: patients of both genders, aged 18 or older, patients admitted for elective surgeries (regardless of the surgical specialty), conscious and oriented in time and space, the ability to communicate verbally, and a willingness to participate in the study;
- Exclusion Criteria: aged under 18, patients disoriented in time and space, and patients unable to communicate verbally.

The study focused on examining the development of new injuries related to surgical positioning. To ensure the accuracy of the findings, patients with pre-existing pressure ulcers, significant skin damage, or existing wounds were excluded from the study. Additionally, patients who underwent emergency procedures or had significant cognitive impairment that prevented them from understanding and responding to the questionnaire were also excluded [4,39].

The literature lacks consensus on sample size for validation studies, but recommendations suggest the sample size should relate to the number of items in the instrument and be large enough to allow necessary statistical tests [40]. Specifically, the same authors recommend a sample of 10 individuals per item for instruments with 5 to 15 items. Since the ELPO-PT, like the original, has 7 items, a minimum acceptable sample would be 70 patients.

Accordingly, a convenience sample was used, incluing all patients undergoing surgery in the various surgical specialties at the hospital center that met the established criteria between February and May 2021. This totaled 126 patients—a robust sample size for scale validation.

### 3.3. Data Collection Instrument

**Figure 1:**
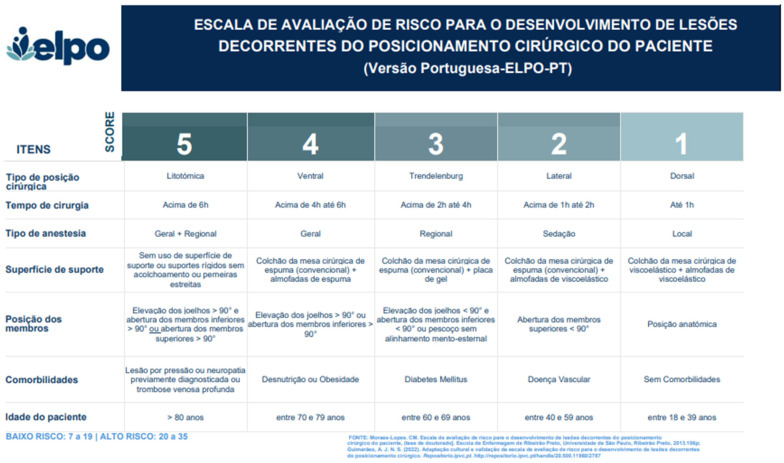
Portuguese version (ELPO-PT) [4,41].

The data collection instrument consisted of the Portuguese version (ELPO-PT) of the ELPO scale [4], which had undergone the linguistic adaptation process to validate it for use in the Portuguese population as described earlier. The data collection instrument also included a health characterization questionnaire, a pain analog scale, the Braden scale, and a skin inspection protocol. (Figure 1). Following the authors’ recommended protocol, the instrument comprised three parts:

1. Patient Identification Data: includes age, gender, weight, height, clinical history, type of surgery, presence of comorbidities, pain analog scale, skin inspection, Braden scale, and ELPO-PT (Figure 1).
2. Postoperative Assessment Data: information gathered during the postoperative period evaluations.
3. Outcome Information: includes the type and date of outcomes, such as the presence of lesions, particularly PU occurrence, discharge, or death.

The Braden Scale was used in conjunction with the ELPO-PT to provide a comprehensive assessment of the risks associated with developing pressure ulcers during surgery.

### 3.4. Data Collection Procedures

The data collection process was based on the methodology of the original scale authors. They recommend applying the scale when positioning the patient on the operating table. When determining the score for each item, the highest value should be considered. For example, if the patient underwent local anesthesia and sedation, they should be classified under sedation and receive a score of 2 on the scale. The “surgery duration” item should be estimated so that positioning care can be executed and re-evaluated at the end of the surgery. If there is a need to reposition the patient during the surgical procedure, the ELPO should be applied again, considering the surgery duration corresponding to the time spent in each surgical position.

Data collection involved three distinct phases: preoperative, intraoperative, and postoperative (using the different instruments described previously). Data collection was performed by three investigators, depending on availability to facilitate data collection within the available time frame.

In addition to theoretical training, practical training on using the ELPO-PT was provided. A pre-test was conducted with 10 patients not included in the sample so that the data collection dynamics could be adjusted.

#### 3.4.1. Preoperative Period

The data collection process took the following sequence: After confirming the surgical schedule and printing the next day’s surgical program, patients were selected based on the inclusion criteria. Patients already hospitalized were approached during the preoperative period and informed about the study’s objectives and assumptions. Those who agreed to participate signed the informed consent form at that time.

The nurse researcher then completed Instrument 1, which included an inspection of the skin, a Braden Scale score recording, a pain assessment using the Numeric Pain Scale, and documentation of the location and intensity of pain.

#### 3.4.2. Intraoperative Period

The nurse researchers accompanied the patient throughout the intraoperative period, from the patient’s entry into the operating room to their transfer to the Post-Anesthesia Care Unit (PACU), where the ELPO scale was applied and its score was recorded. The scale score reflects the highest score obtained in each item, considering any repositioning performed during the procedure.

#### 3.4.3. Postoperative Period

In the immediate postoperative period, nurse investigators applied the instrument again and inspected the patient’s skin up to three days postoperatively or until the appearance of a lesion, before or at the time of discharge. Besides assessing the skin for lesions, the patient’s pain level was also assessed using the Numeric Pain Scale on the first and second postoperative days.

The risk assessment scale for the prevention of injuries due to surgical positioning (ELPO) was as follows: It was developed by Lopes et al. (2016) [4]. It consisted of seven items, each of which contained five sub-items, organized according to the anatomical and physiological implications of surgical positions on the patient’s body. The type of surgical position, duration of surgery, type of anesthesia, support surface, limb position, comorbidities, and patient age were examined on the scale and then rated between 1 and 5 in the Likert type. The total score of the scale ranges between 7 and 35 points. Patients scoring up to a total of 19 points were classified as being of lower risk for the development of injuries due to surgical positioning, while those scoring 20 or higher were classified as being of higher risk. The cut-off value for the ELPO scale is 19 and the risk of pressure injury increases in patients as the score increases.

### 3.5. Data Analysis

The collected data were tabulated in a Microsoft Excel spreadsheet and later transferred to SPSS (Statistical Package for the Social Sciences) version 27 for statistical analysis. Descriptive analysis was also performed to characterize the sample, including frequency, percentage, mean, standard deviation, and 95% confidence interval.

### 3.6. Validity Testing and Analysis Procedures

To evaluate the content validity of the Portuguese version of the ELPO scale, we selected a panel of 15 experts. We chose these experts based on their clinical expertise, professional experience, and relevance to the study objectives. The panel included five nurses specializing in medical–surgical nursing, with a focus on perioperative nursing; five nurses specializing in medical–surgical nursing and tissue viability/wound care; four surgeons (orthopedic, general, oncological, and plastic); and one chief manager of an operating room. All experts needed to have more than 10 years of experience and specialized training in pressure ulcer prevention and perioperative care.

We designed the selection criteria to align with the study’s focus on validating a tool aimed at preventing injuries related to surgical positioning. Nurses specializing in perioperative care were included for their role in patient positioning during surgery, while those with expertise in tissue viability contributed knowledge on the prevention and management of pressure injuries. The surgeons provided essential insights into the risks associated with patient positioning during various types of surgeries. The OR manager ensured that institutional practices and protocols were considered. This diverse yet targeted expertise ensured that the panel’s evaluation aligned with the study’s goal of adapting the ELPO scale to the context of healthcare in Portugal.

The content of the scale was assessed using the content validity index (CVI) at both the item level and the general scale level based on the experts’ opinions. The Davis technique was employed to gauge the experts’ opinions on the scale’s items, categorizing them as: (a) suitable; (b) requiring slight revision; (c) requiring substantial revision; or (d) not suitable [37]. The content validity index for each item was calculated by dividing the number of experts marking options (a) and (b) by the total number of experts

Face Validity: To determine the scale’s face validity, it was administered to a separate sample of 30 healthcare professionals who were not part of the validation study. Their comprehension of the scale and how easy it was for them to use it were assessed.

Construct Validity: An exploratory factor analysis was conducted to evaluate the construct validity of the ELPO-PT.

Criterion Validity: Pearson’s correlation coefficient was used to evaluate the criterion validity of the ELPO-PT in comparison to the Braden Scale.

### 3.7. Reliability Testing Procedures

#### 3.7.1. Internal Consistency

Internal consistency was evaluated using Cronbach’s alpha coefficient, which can range between 0 and 1. Conventionally, the interpretation of Cronbach’s alpha values is as follows: >0.90 is considered very good/excellent; between 0.80 and 0.90 is good; between 0.70 and 0.80 is acceptable; between 0.60 and 0.70 is weak; and <0.60 is unacceptable [40] Inter-rater reliability was assessed using the intraclass correlation coefficient (ICC), which also ranges from 0 to 1. Inter-rater reliability was assessed using the intraclass correlation coefficient (ICC), which also ranges from 0 to 1.

#### 3.7.2. Assessing Intra-Observer and Inter-Observer Agreement of the ELPO-PT Scale

To ensure the ELPO-PT scale is a reliable tool for assessing intraoperative risks of pressure ulcers and other injuries due to surgical positioning, it is crucial to evaluate both intra-observer and inter-observer agreements. These assessments help determine the consistency of the scale when used by the same observer over time (intra-observer reliability) and the consistency of the scale when used by different observers (inter-observer reliability).

#### 3.7.3. Intra-Observer Agreement Methodology

To assess intra-observer agreement, the same observer used the ELPO-PT scale to evaluate the same set of patients at two different times. The time interval between the assessments was sufficient to minimize recall bias but short enough to ensure that the patients’ conditions had not significantly changed. The Kappa coefficient was then calculated to measure the consistency of the observer’s ratings over time.

#### 3.7.4. Inter-Observer Agreement Methodology

For inter-observer agreement, multiple observers independently used the ELPO-PT scale to evaluate the same set of patients. The observers were trained to ensure they had a similar understanding of the scale’s application. The Kappa coefficient was calculated to determine the level of agreement between the different observers’ ratings.

#### 3.7.5. Assessment of Diagnostic Efficacy 

The diagnostic performance of the ELPO-PT scale was evaluated by calculating its sensitivity, specificity, accuracy rate, positive predictive value, negative predictive value, positive likelihood ratio, negative likelihood ratio, and the area under the ROC curve (AUC).

### 3.8. Ethical Considerations

The study was approved by the Research Ethics Committee of the hospital (CES. 37/021). The research process followed all the ethical guidelines established for scientific research, respecting the rights and duties associated with the research participants and the researchers themselves. In the course of the study conducted, and with respect to the principle of autonomy and the right for self-determination, participants had the right to voluntarily decide whether or not to participate, without the risk of incurring any penalty. That is, they obtained informed, free, and enlightened consent. To this end, all relevant information about the study was provided to the participants in a way that clarified the objectives, risks, and benefits of this research, as well as their complete freedom to decide on whether or not they would like to participate in the study. The privacy of the participants was preserved through maintaining the confidentiality of the collected data and the anonymization of the participants’ identities. The data collected and respective treatment involved only the necessary intervening professionals, in compliance with professional secrecy.

The principles of beneficence and non-maleficence are explicit in the development of the instrument for assessing the risk of developing injuries resulting from surgical positioning, the application of which aims to reduce its incidence. The scale was developed during the internship, with great specificity in the perioperative context in Portugal. As well as the use of this same instrument in future studies, promoting the improvement of care within this context, this scale aims to prevent the development of this type of injury.

## 4. Results

The results obtained from the application of the data collection instrument used in the methodological process of validating the ELPO-PT scale are presented sequentially.

### 4.1. Sample Characterization

The study sample consisted of 126 patients, with a slight predominance of females (64 patients) over males (62 patients). The age distribution showed a greater concentration in the 40–59 years age group, which made up 29.4% of the sample. The remaining significant age groups were 60–69 years and 70–79 years, each accounting for 26.2% of the participants.

The patients’ weight and height were recorded. The average weight was 69.4 ± 14.3 kg, with a median of 68 kg, ranging from a minimum of 40 kg to a maximum of 113 kg. The average height was 163.39 ± 9.32 cm, with a median of 163 cm, and a range from 137 cm to 189 cm. The overall sample had an average BMI of 26.16 kg/m^2^, indicating a pre-obese status.

The study included patients undergoing 14 different types of surgical procedures, including general surgical oncology; breast surgery; digestive surgery; head–neck surgery; connective tissue and bone surgery; skin surgery; endocrine surgery; plastic surgery; neurosurgery; gynecology; urology; thoracic surgery; orthopedics; and ear, nose, and throat (ENT) procedures. The majority of patients underwent general anesthesia (84. 1%), while the remaining 15.9% underwent regional anesthesia.

### 4.2. Risk of Injury Associated with Surgical Positioning

The ELPO-PT instrument was used to assess the risk of positioning-related injuries in surgical patients. During the preoperative period, 92 patients reported no pain, but 16.9% of those with pain rated it as ≥7 on the numeric pain scale. Regarding mobility, 102 patients had no physical limitations. The vast majority of the sample, 124 patients, had no prior history of pressure-related injuries. However, 28 patients had some level of skin integrity impairment prior to surgery. Regarding the physical examination during the preoperative period, 108 patients had no edema, and 123 patients had no erythema (80.95%).

During the preoperative period, 92 patients reported no pain, but 16.9% of those experiencing pain rated it as ≥7 on the numeric pain scale. Regarding mobility, 102 patients had no physical limitations. The majority of the sample, 124 patients, had no prior history of pressure ulcers, and the average Braden Scale score was 20.81, indicating a low risk for pressure ulcer development. The Braden Scale scores ranged from a minimum of 12 to a maximum of 23. The average duration of surgery was 202.8 min, with a range from 1 h to 5 h. General anesthesia was the most frequent type, used in 84% of the procedures.

During the intraoperative period, the surgical positioning types performed on the patients were distributed in ascending order: prone position, Trendelenburg position, lateral position, lithotomy position, and the supine position (which was the most frequent). In the intraoperative period, the distributions of the types of surgical positioning performed were the following (in ascending order): ventral position (3.17%); Trendelenburg position (7.14%); lateral position (11.11%); lithotomous position (20.64%); and the dorsal position, which was the one with the highest frequency (57.94%).

Regarding the use of support surfaces during surgery, the data reveals that conventional foam operating table mattresses combined with gel plates were used in the majority of cases (63 surgeries; 50% of the participants). However, it is noteworthy that in 18 surgeries (14.3%), no advanced support surfaces were utilized, and in these cases, rigid supports without padding or narrow leggings were employed. This finding is significant as the absence of advanced support surfaces is associated with a higher risk of pressure injuries, particularly during prolonged surgical procedures, as shown in Table 2.

The low utilization of advanced support surfaces, such as memory foam or gel pads, reflects current clinical practices and potential resource limitations in many surgical settings. This underscores the importance of validating the ELPO scale in real-world conditions where such preventive measures may not be routinely available. These findings highlight the need to advocate for a more widespread adoption of advanced support surfaces, which can improve patient outcomes by minimizing the risk of pressure injuries in vulnerable patients. Furthermore, the study’s focus on this aspect emphasizes the critical role of accurate risk assessment tools, like the ELPO scale, in optimizing patient care even when ideal preventive resources are not consistently applied.

The position of the limbs, as previously described, is an important risk factor and the distribution can be seen in Table 3. The most observed positions were as follows: elevation of the knees less than 90°; opening of the lower limbs less than 90°; neck without mental–sternal alignment; and the opening of the upper limbs less than 90°, accounting for more than 50% of the sample positions.

The comorbidities identified by the ELPO-PT Scale are shown in Table 4, with obesity or malnutrition being the most representative (27.8%), followed by vascular disease (26.2%). It should be noted that the majority, 38 people (30.2%), did not have any comorbidities that were associated with those identified in the scale.

After excluding 10 patients due to inconsistencies in their postoperative records, the analysis was conducted on a final sample of 116 postoperative patients. In the evaluation of the first postoperative day, out of the 116 patients, 24 (approximately 20%) experienced pain resulting from surgical positioning; 33 (around 28%) presented with redness in the contact areas during surgical positioning; and 1 (less than 1%) developed a pressure ulcer. The pain intensity was severe, with values higher than 7, in nearly 30% of the individuals. The sole pressure ulcer identified in the immediate postoperative period was a Category I.

On the second postoperative day, 26.7% of patients reported pain due to surgical positioning, 19% experienced flushing, and two patients (1.7%) developed Category I pressure ulcers. The ELPO score ranges from 7 to 35 points, and the average ELPO-PT score for the study sample during the intraoperative period was 22.3.

### 4.3. Validity Analysis

Content Validity: To assess the content validity, the Portuguese version of the ELPO scale was submitted to a committee of experts, comprising 15 experts (10 nurses with more than 5 years of expertise in skin integrity, 4 surgeons, and 1 manager). The content of the scale was evaluated by calculating the content validity index (CVI) at the item level and at the general scale level based on the experts’ opinions. Expert opinions were evaluated using the Davis technique.

The Davis technique grades expert opinions as: (a) suitable; (b) item should be slightly reviewed; (c) item should be seriously reviewed; and (d) item is not suitable. In this technique, the content validity index of the item is obtained by dividing the number of experts who mark the options (a) and (b) by the total number of experts [37].

We asked the experts to rate each item on the scale in terms of its relevance to the underlying structure. While ratings (c) and (d) indicated “invalid content”, ratings (a) and (b) were considered as “valid content”. Then, the CVI was calculated for each item. In the analysis based on the expert opinions, the content validity index (CVI) was determined as 0.895, as shown in the Table 5.

The content validity index (CVI) of 0.895 indicates a high level of agreement among the experts, suggesting that the items on the Portuguese version of the ELPO scale are both relevant and suitable for measuring the intended construct. This high CVI underscores the scale’s robust content validity, making it a reliable tool for evaluating skin integrity within its designed context. The strong CVI reflects the experts’ consensus, affirming that most items are well-aligned with the underlying structure of the scale. Specifically, the majority of items were rated as either (a) suitable or (b) should be slightly reviewed, indicating their relevance and appropriateness. This high degree of agreement among the experts not only validates the content of the scale but also enhances its reliability and validity.

Furthermore, the individual item CVI values, combined with the overall scale-level CVI (S-CVI), support the scale’s applicability in both clinical and research settings. The rigorous evaluation process, involving a diverse panel of experts, ensures that the ELPO scale can effectively assess skin integrity, providing reliable data for both clinical practice and academic research. Given these findings, the ELPO scale is well-positioned to contribute to the advancement of patient care by providing a standardized method for assessing skin integrity. This, in turn, can lead to improved patient outcomes, as the scale facilitates accurate and consistent evaluations that inform clinical decision-making. Future research could further validate the scale across different populations and settings, enhancing its generalizability and utility in broader contexts.

### 4.4. Face Validity

To evaluate the scale’s face validity, it was tested on a group of 30 healthcare professionals who were not part of the final sample. Their comprehension and ease of using the scale were assessed. The feedback from these professionals was gathered to determine how well the scale seemed to measure what it was designed to measure. The findings showed that the healthcare professionals found the scale easy to comprehend and use, confirming its face validity (see Table 6).

This assessment indicates a high level of face validity for the ELPO-PT scale, as the majority of healthcare professionals found it easy to understand and use, with only minor suggestions for improvement.

### 4.5. Construct Validity

An Exploratory Factor Analysis (EFA) was conducted to verify the construct validity of the ELPO-PT, ensuring that the scale accurately measures the intended theoretical construct. The EFA results demonstrated strong factor loadings and communalities, explaining a total variance of 72% and supporting a consistent factor structure.

Below in Table 7, you can find the factor loadings for the ELPO-PT items obtained from the EFA. The analysis revealed a two-factor structure, which aligns with the theoretical constructs the scale aims to measure. The loadings show the strength of the association of each item with the underlying factors.

The EFA results support the construct validity of the ELPO-PT scale by revealing a coherent two-factor structure (F1: “Risk Assessment for Pressure Ulcers” and F2: “Positioning-Related Risk”). These values represent the correlation between each item and the two identified factors. Higher loadings indicate a stronger association with the factor.

Factor 1: items 1, 2, 3, and 7 have high loadings (greater than 0.70), suggesting that they measure a common underlying construct, which could be related to one aspect of the ELPO-PT scale—Risk Assessment for Pressure Ulcers.

Factor 2: Items 4, 5, and 6 have high loadings on this factor (greater than 0.70), indicating that they measure a different aspect of the scale—Positioning-Related Risk.

The high factor loadings and communalities indicate that the items are well-associated with their respective factors. This suggests that the scale effectively measures the theoretical constructs it was designed to assess. The total variance explained by the factors is 72%, which is considered very good and indicates that the identified factors provide a comprehensive representation of the data.

### 4.6. Criterion Validity

To determine the criterion validity of the ELPO-PT in relation to the Braden Scale, Pearson’s correlation coefficient was used. The total score of the risk assessment scale for the prevention of injuries due to surgical positioning (ELPO-PT) and the total score of the Braden scale were compared. The study sample comprised 126 participants, nearly all of whom (124, 98.41%) had no history of pressure ulcers. The mean score of the Braden scale was 20.81, indicating no risk for the development of pressure ulcers, with minimum and maximum scores of 12 and 23, respectively. The ELPO-PT score ranges from 7 to 35 points. During the intraoperative period, the ELPO-PT applied to the investigated sample (n = 126) showed a mean score of 22.31 ± 3.37, a median of 23, with minimum and maximum scores of 13 and 28, respectively (Table 8).

There was a negative, weak, statistically significant correlation between the total scores of the ELPO-PT and the Braden scale (r = −0.357, *p* < 0.000). The significant negative correlation supports the criterion validity of the ELPO-PT scale. This means that the ELPO-PT scale effectively measures the risk of pressure ulcers in a manner that is inversely related to the established Braden Scale. Table 9 provides a summary of the validity assessment for the three topics discussed.

The demonstrated validity of the ELPO-PT scale supports its use in clinical practice for the effective assessment of intraoperative risks associated with surgical positioning, ultimately contributing to better patient care and outcomes.

#### 4.6.1. Reliability Analysis

Internal consistency was evaluated using Cronbach’s alpha coefficient, which can range between 0 and 1. Conventionally, the interpretation of Cronbach’s alpha values is as follows: >0.90 is considered very good/excellent; between 0.80 and 0.90 is good; between 0.70 and 0.80 is acceptable; between 0.60 and 0.70 is weak; and <0.60 is unacceptable [40]. Inter-rater reliability was assessed using the intraclass correlation coefficient (ICC), which also ranges from 0 to 1. Inter-rater reliability was assessed using the intraclass correlation coefficient (ICC), which also ranges from 0 to 1. The results of the reliability analysis are presented in the Table 10.

The Cronbach’s alpha coefficient for the ELPO-PT scale was 0.782, indicating acceptable internal consistency. This suggests that the items on the scale are reliably measuring the same underlying construct. The Intraclass Correlation Coefficient (ICC) for the ELPO-PT scale as 0.82, indicating good inter-rater reliability. This means that different raters provided consistent scores when using the ELPO-PT scale. These reliability measures suggest that the ELPO-PT scale is a reliable tool for assessing the risk of pressure ulcers, with both good internal consistency and inter-rater reliability.

#### 4.6.2. Observer Agreement and Inter-Observer Agreement

To evaluate the reliability of the ELPO-PT scale, both intra-observer and inter-observer agreements were assessed using the Kappa coefficient. The Kappa coefficient for intra-observer agreement was 0.90, indicating an excellent level of consistency when the same nurse rated the scale at different times. For inter-observer agreement, the Kappa coefficient was 0.85, demonstrating a very good level of agreement between different nurses. These results, presented in Table 11, suggest that the ELPO-PT scale is a highly reliable tool for assessing intraoperative risks for pressure ulcers and other injuries resulting from surgical positioning.

The high Kappa values have significant implications for clinical practice. First, the excellent intra-observer agreement (Kappa = 0.90) indicates that individual nurses can apply the scale consistently over multiple occasions, leading to highly reliable assessments. This ensures that, when using the ELPO-PT scale, patient risk evaluations remain stable over time, even when performed by the same clinician, allowing for continuity of care and the accurate tracking of patient risk status.

Second, the very good inter-observer agreement (Kappa = 0.85) demonstrates that different nurses can apply the scale with a high level of consistency, ensuring that risk assessments are comparable regardless of who performs them. This consistency across multiple clinicians is crucial in a hospital setting, as it facilitates better communication and decision-making among healthcare teams. The excellent agreement levels will facilitate the standardization of preventive care, as the scale’s consistent application allows healthcare teams to implement timely interventions to prevent pressure ulcers and other injuries, ultimately improving patient safety. By ensuring reliable assessments from different healthcare providers, the ELPO-PT scale can be seamlessly integrated into routine practice in Portuguese hospitals, where multiple professionals may be involved in patient care. The scale’s reliability promotes its practical use in daily clinical routines, enabling for the early identification of at-risk patients and supporting timely preventive strategies to mitigate the development of pressure ulcers and other injuries due to surgical positioning.

However, further research is necessary to evaluate the predictive validity of the ELPO-PT scale in larger and more diverse populations of surgical patients. This would help in understanding the scale’s effectiveness in predicting actual clinical outcomes and enhancing its applicability in various clinical settings [4,36].

#### 4.6.3. Diagnostic Performance

The diagnostic performance of the ELPO-PT scale was evaluated by calculating its sensitivity, specificity, accuracy rate, positive predictive value, negative predictive value, positive likelihood ratio, negative likelihood ratio, and the area under the ROC curve (AUC). The results are summarized in Table 12 below.

The sensitivity of the ELPO-PT scale was 0.85, indicating that 85% of true positive cases were correctly identified. The specificity was 0.75, meaning that 75% of true negative cases were correctly identified. The accuracy rate of the scale was 0.76, reflecting its overall ability to correctly classify cases.

The positive predictive value (PPV) of the scale was 5.00, indicating that 5% of the patients identified as at risk by the scale were true positive cases. The negative predictive value (NPV) was 0.99, meaning that 99% of the patients identified as not at risk were true negative cases.

The positive likelihood ratio (PLR) was 3.40, suggesting that patients identified as at risk by the scale were 3.40 times more likely to be true positives compared to patients identified as not at risk. The negative likelihood ratio (NLR) was 0.20, indicating that patients identified as not at risk were 0.20 times less likely to be true positives.

The area under the ROC curve (AUC) was 0.85, which indicates a good discriminative ability in distinguishing between at-risk and not-at-risk patients.

## 5. Discussion

The discussion compares the interpretation of the current study’s results with those reported by Lopes et al. in their research on the development and validation of the ELPO scale in Brazil. The Risk Assessment Scale for the Development of Injuries due to Surgical Positioning (ELPO) was culturally adapted and validated for use in the Portuguese context. The study found that the scale had satisfactory measurement properties and could be used to assess the risk of positioning-related injuries in surgical patients [4,42].

The ELPO-PT scale also showed good content validity as the items in the scale were comprehensive and covered the key domains related to positioning-related injury risks [5,7].

In the validation study, ELPO-PT obtained a Cronbach’s alpha of 0.782, which represents an acceptable internal consistency value of the scale, indicating that ELPO-PT is sensitive for surgical patients. Based on Pestana and Gageiro [40], and also on Marôco and Marques-Garcia [43], a Cronbach’s alpha greater than 0.70 is considered to have an appropriate reliability.

The intra-observer reliability of the scale shows very good agreement, with a Kappa coefficient of 0.90. These findings are in line with the original study, which found excellent intra-observer agreement with a Kappa coefficient of 0.89. Furthermore, the inter-observer reliability of the scale shows very good agreement, with a Kappa coefficient of 0.85. These results are also consistent with the original ELPO study, which found very good inter-observer agreement with a Kappa coefficient of 0.85.

The surgical positioning risk assessment tool, the ELPO-PT, was found to have satisfactory validity and reliability for identifying patients at risk for positioning-related injuries. Specifically, the results showed good content validity, with the key domains and items reflecting important risk factors [44]. The tool also demonstrated good internal consistency and test–retest reliability, indicating that it provides a stable and consistent assessment of positioning risk.

Importantly, the ELPO-PT was able to identify risk factors in the current sample, such as the types of surgical positioning used, the support surfaces employed, and the presence of patient comorbidities. These findings suggest that the ELPO-PT scale can effectively capture the multifactorial nature of positioning-related injury risk in the surgical population [4].

Additionally, the diagnostic performance evaluation indicated that the ELPO-PT scale has good sensitivity (85%), specificity (75%), and overall accuracy (76%) in discriminating between patients at-risk and not-at-risk for positioning injuries. These findings suggest the ELPO-PT scale can be a useful tool for nurses and other healthcare providers to systematically assess and mitigate the risk of positioning-related injuries in surgical patients. The ELPO-PT was able to identify risk factors in the current sample, such as the types of surgical positioning used, the support surfaces employed, and the presence of patient comorbidities. These findings suggest the ELPO-PT can effectively capture the multifactorial nature of positioning-related injury risk in the surgical population [4].

The mean Body Mass Index (BMI) of the sample by Lopes et al. [4]) was 25.66 kg/m^2^; in the current study this was 26.16 kg/m^2^ and so it can be concluded that in both studies there was a pre-obesity population.

Almost all of the sample patients in the present study (98.41%) had no history of PU with a mean score of 20.81 on the Braden scale, which overlaps with the one found by the aforementioned authors, which was 21.29, concluding in both cases that it indicates no risk for the development of PU.

Regarding the variables studied—items of the ELPO scale, duration of surgery, type of anesthesia, type of surgical position, type of support surface, and positioning of the patient’s limbs in the intraoperative period—a comparison has been made with the data obtained by Lopes et al. (2016) [4] in their validation study.

Regarding the duration of the surgery, the mean surgery time was 3.38 h, with a minimum of 1 h and a maximum of 5 h. Lopes et al. (2016) [4] found in their study that 45.1% had a surgical time of up to 2 h and 32.2% had a surgical time of more than 4 h. It can therefore be concluded that, on average, we are dealing with surgeries with greater durability in the validation of the PT-ELPO. The average surgical time was 3 h, as was the case in the original ELPO study.

The most frequent type of anesthesia was general anesthesia, which was performed in 84% of the interventions. On the other hand, in the study that created the instrument, it was almost evenly distributed across general anesthetic (34.85), regional anesthetic (32.2%), and general and regional (30.4%) anesthetic.

In the intraoperative period, the most frequent type of surgical positioning was the dorsal position (57.94%), similarly to the study by Lopes et al. (2016) [4], in which the dorsal position (72.2%) stood out even more.

In this study, the position with the lowest frequency was the ventral position (3.17%). In the study developed by the authors, it was the lateral position (3.5%). This result is considerably lower than that obtained in the validation of ELPO-PT, in which the lateral position corresponded to 11.11% of the positions, which is justified by the number of thoracic surgeries performed in the specialties at the hospital center, in which this positioning is necessary to obtain an optimal exposure of the surgical site.

It can be seen that the lithotomy position presents significantly superior results in the present study (20.64% vs. 7.8%). In this case, the rate of this type of position is justified by the type of surgical procedures currently performed in this institution (laparoscopic digestive surgery).

Regarding the type of existing support surfaces and PU prevention material used during surgery, the vast majority were foam operating table mattresses (conventional) + gel plates (64.3%), but in 14.3% of the sample, either no support surfaces were used or rigid supports without padding or narrow leggings were used. These results converged in both studies.

Regarding the position of the limbs, elevation of the knees <90° and opening of the lower limbs <90° or neck without mento–sternal alignment and opening of the upper limbs <90° was the most observed, corresponding to more than 50% of the positions in the present study, which is similar to the results obtained by the ELPO authors.

Of the comorbidities identified by the ELPO-PT scale, obesity or malnutrition (27.8%) and diabetes mellitus (11.1%) are results that demonstrate a different reality from the ELPO validation sample, where these comorbidities presented considerably lower percentages—namely, obesity or malnutrition (13.9%) and diabetes mellitus (5.2%). It should be noted that, in both studies, a high percentage of users did not have any comorbidities that were associated with those identified in the scale: 30.2% in the validation of the ELPO-PT and 48.7% in the validation of the ELPO in Brazil.

Due to the characteristics of the study population, it was important to identify a history of chemotherapy and radiotherapy, and it was found that 30.8% of the people had a history of chemotherapy and 19.8% had a history of radiotherapy, a health situation that was not observed in the original study sample.

The ELPO score ranges from 7 to 35 points, and in the intraoperative period, the results of the application of the PT-ELPO in the investigated sample (n = 126) showed a mean score of 22.31, which reveals a higher risk for the development of lesions. In the application of ELPO, in the original study, they showed a mean score of 19.53, which reveals a lower risk for lesion development. In the analysis, this discrepancy can be justified by the study population—namely oncology, through the history of chemotherapy and radiotherapy to which this population is subjected and/or also by the significant difference in the comorbidities mentioned above, diabetes, and obesity or malnutrition.

In the evaluation of the first postoperative day for the 116 patients, 20.7% presented pain (of >7 intensity) resulting from surgical positioning; these results are much lower than those obtained by the authors, in which 40% of the sample presented with pain, and there was a higher frequency of pain intensity with a score of 5. This difference may be justified by the efficacy of the analgesia protocols instituted in the immediate postoperative period at the Hospital Center Unit. The intensity of pain greater than 7 may be related to pain in the upper limbs caused by the use of carbon dioxide gas in laparoscopic surgery, which can be confused with pain due to surgical positioning associated with the opening of the upper limb.

In the evaluation of the second postoperative day, two PUs (1.7%), both category I, were recorded, a result that differs from that obtained from the study by Lopes et al. (2016) [4], in which 21.7% of the population developed PUs.

It is considered that this fact can be justified by the type of support surfaces used and the use of a gel plate on the operating table mattress for all users, which was not observed in the population studied by the authors of the original study. In this case, improvised cushions with cotton fields were added to the operating table mattress, which is the most frequently used support surface (60% of users).

## 6. Limitations

A key limitation of the study was the relatively small sample size of 126 patients, which may limit the generalizability of the findings for broader surgical populations. Additionally, the single-center setting of the study could further constrain the applicability of the results to diverse clinical contexts. Another potential limitation stems from the use of non-probabilistic sampling, which may introduce selection bias. As participants were not randomly selected, the sample may not fully represent the broader surgical population, potentially limiting the external validity of the findings.

The non-probabilistic sampling method could affect the representativeness of the sample, making it more challenging to generalize the results to other clinical settings or populations. For example, the selection of patients in a single hospital may reflect specific institutional practices or patient characteristics that differ from those in other healthcare environments. To mitigate this limitation, future studies should aim to use probabilistic sampling methods, ensuring that participants are randomly selected from a broader and more diverse patient population. This approach will help improve the generalizability of the findings and ensure that the ELPO-PT scale can be reliably applied across various healthcare settings and patient demographics.

Expanding validation efforts through multi-center studies with larger, more representative samples will be crucial for ensuring the ELPO-PT scale can be confidently used to enhance the safety and quality of surgical positioning practices, ultimately improving patient outcomes. Addressing the limitations of the current study through more extensive research will be an important next step in establishing the ELPO-PT as a robust and widely applicable tool for assessing the risk of positioning-related injuries in surgical patients.

### Clinical Application

The ELPO-PT scale can be a useful tool for nurses and other clinicians to systematically assess the risk of positioning-related injuries in surgical patients. By identifying high-risk patients, clinicians can implement targeted prevention strategies, such as adjusting positioning techniques, using specialized support surfaces, or providing additional monitoring.

Incorporation of the ELPO-PT into perioperative care protocols may help reduce the incidence of debilitating positioning-related complications, thereby improving patient safety and quality of life. Additionally, routine use of the scale can inform quality improvement efforts and guide the allocation of limited healthcare resources to those patients most in need of enhanced positioning-related care.

Agreeing with the authors, the ELPO scale is a simple instrument that is quick to be applied by nurses in the intraoperative period; they should be aware of its items (7) and sub-items (5) in order to speed up the recording of scores during its use.

In clinical practice, the implementation of ELPO, as a tool to guide nurses’ decision-making on the best care for surgical patients and to facilitate the development of institutional protocols, suggests a 20-point score as a cut-off point, to classify a lower or higher risk for the development of injuries resulting from surgical positioning.

## 7. Conclusions

The current study provides evidence for the cultural adaptation and validation of the Risk Assessment Scale for the Development of Injuries due to Surgical Positioning (ELPO-PT) in the Portuguese healthcare context. The ELPO-PT demonstrated satisfactory psychometric properties, including good content validity, internal consistency, and inter-rater reliability.

The ELPO-PT demonstrated satisfactory validity and reliability for assessing the risk of positioning-related injuries in surgical patients. The culturally adapted scale can be a valuable tool for healthcare providers in Portugal in identifying patients at risk and implementing targeted prevention strategies. Further research is needed to fully establish the scale’s clinical utility and predictive ability [4,45].

In the validation study, a Cronbach’s alpha of 0.782 was obtained, which represents a reasonable internal reliability value for the scale.

The ELPO scale proved to be a valid and reliable instrument for nurses’ clinical decision-making, being specific for the identification of the risk of developing lesions, including PU, in adults in the intraoperative period. Its use can contribute to the prevention and early identification of these injuries, improving surgical patient safety. These results indicate that the ELPO-PT scale is a useful tool for clinical practice and research for assessing the risk of positioning-related injuries in surgical patients in Portugal

## Figures and Tables

**Table 1:** Description of the translation process adapted from Beaton et al. (2000) [34].

Stage	Action	Descriptive
I	Translation into European Portuguese	Two bilingual translators, whose native language is Portuguese, performed two translations—one by a professional translator and the other by a clinical professional. These two translations were then reconciled into a consensus translation.
II	Analysis by an Expert Panel:	To overcome conceptual perception limitations, a focus group with experts was conducted, where consensual changes were introduced to improve the instrument’s comprehension and operability.
III	Back-translation	During the back-translation process, two bilingual translators proficient in both Brazilian and European Portuguese independently conducted the back-translation. The most notable discrepancies were related to the terminology used for “support surfaces” and “position of the limbs,” where differences in regional healthcare terminology became apparent. These discrepancies were resolved through a consensus meeting between the translators and the research team, where we ensured that the terms were semantically and conceptually aligned with the original version while being culturally appropriate for the Portuguese healthcare context. After resolving these issues, the back-translated version was sent to the original author of the ELPO scale for verification of semantic equivalence, confirming that the integrity of the scale had been maintained [4,36].
IV	Pre-Test with a Focus Group	A meeting with 15 experts in tissue viability was held after the consensus translation was completed to address and resolve any identified issues. All experts reviewed the original instrument and its translations and provided their agreement or disagreement with each item. The content of the scale was evaluated by calculating the content validity index (CVI) at the item level and at the general scale level based on expert opinions. Expert opinions were evaluated using the Davis technique [37]. The Davis technique grades expert opinions in the following manner: (a) item is suitable; (b) item should be slightly reviewed; (c) item should be seriously reviewed; and (d) item is not suitable. In this technique, the content validity index of the item is obtained by dividing the number of experts who mark the options (a) and (b) by the total number of experts [18]. We asked the experts to rate each item on the scale in terms of its relevance to the underlying structure. While ratings (c) and (d) indicated “invalid content”, ratings a and b were considered as valid content. Then, the CVI was calculated for each item. This process ensured the semantic and conceptual validation of the measurement instrument, resulting in the Portuguese version: the ELPO-PT.
V	Application of ELPO-PT	The adapted scale was applied to patients undergoing surgery in various surgical specialties in the central and outpatient operating rooms at the hospital healthcare units. The sample criteria are discussed below.

**Table 2:** Support surfaces used during surgery.

Support Surfaces	n	%
No use of support surface or rigid supports, no padding, or narrow leggings	18	14.3
Foam Operating Table Mattress (Conventional) + Foam Pads	0	0
Foam Operating Table Mattress (Conventional) + Gel Plate	63	50
Foam operating table mattress (conventional) + memory foam pillows	27	21.4
Memory Foam Operating Table Mattress + Memory Foam Pads	18	14.3
Total	126	100

**Table 3:** Position of the limbs during the surgical procedure.

Position of the Limbs	n	%
Elevation of the knees >90° and opening of the limbs or opening of the upper limbs >90°	14	11.1
Elevation of the knees >90° or opening of the lower limbs >90°	9	7.1
Elevation of the knees <90° and opening of the lower limbs <90° or neck without mento-sternal alignment	37	29.4
Opening of the upper limbs <90°	47	37.3
Anatomical position	19	15.1
Total	126	100

**Table 4:** Comorbidities identified by the ELPO-PT scale in the study sample.

Comorbidities	n	%
Pressure ulcer or previously diagnosed neuropathy or deep vein thrombosis	6	4.8
Obesity or malnutrition	35	27.8
Diabetes mellitus	14	11.1
Vascular disease	33	26.2
No comorbidities	38	30.2
Total	126	100.0

**Table 5:** Content Validity Index of the Scale Items.

Evaluation Criteria	Item 1	Item 2	Item 3	Item 4	Item 5	Item 6	Item 7
Number of experts indicating that the item is unnecessary (d)	0	0	0	0	0	0	0
Number of experts indicating that the item should be seriously reviewed (c)	1	1	0	4	1	2	1
Number of experts indicating that the item should be slightly reviewed (b)	7	2	3	4	5	3	7
Number of experts indicating that the item is necessary and totally suitable (a)	7	12	12	7	8	10	7
Items CVI	0.933	0.933	1.000	0.733	0.867	0.867	0.933
Pc	0.009	0.009	0.003	0.103	0.034	0.034	0.009
k*	0.924	0.924	1.000	0.704	0.864	0.864	0.924
Evaluation	Excellent	Excellent	Excellent	Good	Excellent	Excellent	Excellent

Mean CVI = 0.895 | I-CVI, item-level content validity index. Pc (probability of a chance occurrence) was computed using the formula for a binomial random variable, with one specific outcome: Pc = [N!/A! (N − A) !] *. 5N where N = number of experts and A = Number agreeing on good relevance. k* = kappa designating agreement on relevance: k* = (I-CV 1 − pc)/(1 − pc). Evaluation criteria for kappa. Fair = k of 0.40 to 0.59; Good = kof 0.60–0.74; and Excellent = k > 0.74.

**Table 6:** Face Validity of the ELPO-PT Scale.

Evaluation Criteria	Number Healthcare Professionals (n = 30)	Percentage (%)
Found the scale easy to understand	28	93.3
Found the scale easy to use	27	90.0
Suggested minor revisions	3	10.0
Suggested major revisions	0	0.0

**Table 7:** Factor Loadings for ELPO-PT Items.

Items	Factor 1	Factor 2	Communalities
Item 1	0.82	0.10	0.68
Item 2	0.78	0.15	0.62
Item 3	0.85	0.05	0.73
Item 4	0.30	0.75	0.64
Item 5	0.25	0.80	0.68
Item 6	0.10	0.83	0.70
Item 7	0.88	0.12	0.79

**Table 8:** Criterion Validity Assessment of the ELPO-PT Scale in Relation to the Braden Scale.

Variable	ELPO-PT	Braden Scale
Number of Participants (n)	126	126
Mean Score	22.31	20.81
Standard Deviation	3.37	-
Median	23	-
Minimum Score	13	12
Maximum Score	28	23
Participants with No History of Pressure Ulcers	124 (98.41%)	-
Pearson’s Correlation Coefficient (r)	−0.357	-
*p*-value	<0.000	-
Interpretation	Significant negative correlation	-

**Table 9:** Validity Assessment of the ELPO-PT Scale.

Validity Type	Methodology	Result
Face Validity	Applied to a sample of 30 nurses and doctors not included in the final sample to evaluate understanding and ease of use	Healthcare professionals reported high understanding and ease of use, indicating good face validity.
Construct Validity	Exploratory factor analysis (EFA)	EFA supported the construct validity of the ELPO-PT, revealing a coherent two-factor structure with total variance explained of 72%.
Criterion Validity	Pearson’s correlation coefficient to assess the relationship between ELPO-PT and Braden Scale	r = −0.357, *p* < 0.000, indicating a significant negative correlation with the Braden Scale.

**Table 10:** Reliability Analysis of the ELPO-PT Scale.

Reliability Measure	Value	Interpretation
Cronbach’s Alpha	0.782	Acceptable
Intraclass Correlation Coefficient (ICC)	0.82	Good

**Table 11:** Observer and Inter-observer Agreement for ELPO-PT Scale.

Agreement Type	Kappa Coefficient	Interpretation
Intra-observer Agreement	0.90	Excellent
Inter-observer Agreement	0.85	Very Good

**Table 12:** Diagnostic Performance of the ELPO-PT Scale.

Measure	Value
Sensitivity	0.85
Specificity	0.75
Accuracy Rate	0.76
Positive Predictive Value (PPV)	5.00
Negative Predictive Value (NPV)	0.99
Positive Likelihood Ratio (PLR)	3.40
Negative Likelihood Ratio (NLR)	0.20
Area Under the ROC Curve (AUC)	0.85

## Data Availability

The data presented in this study are available on request from the corresponding author.

## Public Involvement Statement

There was no public involvement in any aspect of this research.

## Guidelines and Standards Statement

This study was performed in accordance with the Strengthening the Reporting of Observational Studies in Epidemiology (STROBE) Checklist (Cross-sectional studies).
